# The Role of Testin in Human Cancers

**DOI:** 10.1007/s12253-018-0488-3

**Published:** 2018-10-25

**Authors:** Aneta Popiel, Christopher Kobierzycki, Piotr Dzięgiel

**Affiliations:** 1 Division of Histology and Embryology, Department of Human Morphology and Embryology, Wroclaw Medical University, Wroclaw, Poland; 2 Wroclaw Medical University, Wroclaw, Poland

**Keywords:** Testin, Cancerogenesis, Breast cancer, Ovarian cancer, Gastrointestinal cancers

## Abstract

Testin is a protein expressed in almost all normal human tissues. It locates in the cytoplasm along stress fibers being recruited to focal adhesions. Together with zyxin and vasodilator stimulated protein it forms complexes with various cytoskeleton proteins such as actin, talin and paxilin. They jointly play significant role in cell motility and adhesion. In addition, their involvement in the cell cycle has been demonstrated. Expression of testin protein level correlates positively with percentage of cells in G1 phase, while overexpression can induce apoptosis and decreased colony forming ability. Decreased testin expression associate with loss by cells epithelial morphology and gain migratory and invasive properties of mesenchymal cells. Latest reports indicate that *TES* is a tumor suppressor gene which can contribute to cancerogenesis but the mechanism of loss *TES* gene expression is still unknown. Some authors point out hypermethylation of the CpG island as a main factor, however loss of heterozygosity may also play an important role [4, 5]. The altered expression of testin was found in malignant neoplasm, *i.a.* ovarian, lung, head and neck squamous cell cancer, breast, endometrial, colorectal, prostate and gastric cancers [1–9]. Testin participate in the processes of tumor growth, angiogenesis, and metastasis [10]. Many researchers stated involvement of testin in tumor progression, what suggest its potential usage in immunotherapy [7, 11]. Understanding the molecular functions of testin may be crucial in development personalized treatment. In the present manuscript up-to-date review of literature can be found.

## Introduction

### Testin in Physiology

**Fig. 1:**
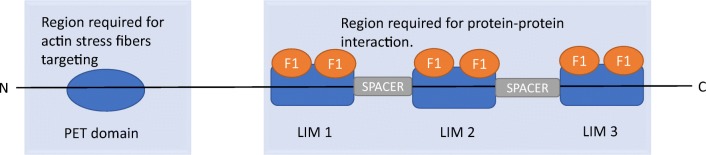
The structure of the testin protein

Testin is a protein with a molecular mass 47 kDa encoded by *TES* gene located on the fragile site FRA7G at 7q31.2 [6]. It is localized in the cytoplasm, along stress fibers and is recruited to focal adhesions [12]. The protein is composed of N-terminal PET (Prickle, Espinas, *TES*) and C-terminal three LIM (lin–11, isl-1, mec-3) domains [13]. At its COOH terminal (Fig. 1), the testin protein has three zinc-binding domain linked by two amino-acids spacer which play role in focal adhesion. LIM family proteins have been found to be a part of cytoskeleton [14]. They are responsible for protein-protein interactions coordinating signaling intracellular pathways [15, 16]. The N-terminal and C-terminal halves of the protein can interact with each other, hence hindering interaction with other cytoskeleton associated protein such as: zyxin, vasodilator stimulated protein (VASP), talin, nuclear actin related protein (Arp7A) and actin [17]. They are together acting as integrating partners in focal adhesion. Coutts et al. noticed that fibroblasts stably overexpressing TES have an increased ability to spread, are larger and contain increased numbers of actin protrusions [16]. Testin protein overexpression had effect on cells spreading potential not on percentage of cells spread. In view of described built, testin plays significant role in the cell adhesion, cell spreading and in the reorganization at the actin cytoskeleton [5, 18, 19].

### Testin in Pathology

Molecular studies characterized *TES* as tumor suppressor gene and reported its downregulation in many human malignancies [1–3, 6–8, 20]. In 2007 Boëda et al. presented for the first time interaction between LIM3 domain of testin protein and Mena protein which is key modulator of cellular migration [18]. Moreover, it was shown, that decreased expression of testin protein increased cell motility, decreased cell-cell contact and therefore have potential to be a marker of cancer metastasis [4, 21, 22].

TES encodes testin protein containing a PET domain at the NH2- terminus which is involved in actin stress fibers targeting, and three LIM ((lin-1, ils-1 and mec-3) domains (LIM1, LIM2, LIM3) at the COOH-terminus. One LIM domain contain loosely conserved cysteine-rich consensus sequence including two separate zinc fingers-F1. They are separated from each other by SPACER.

Cancers are the only investigated diseases with *TES* gene disruptions. It is silencing promotes cell proliferation, invasiveness ability and angiogenesis [23, 24]. Important ways of *TES* inactivation are mechanisms of loss of heterozygosity (LOH) and hypermethylation (HMT). In LOH one or two alleles of the same gene are lost, whereas in HMT occurs abnormal DNA methylation which may inactivate suppressor genes. This phenomena were described in almost every type of cancer. Predominantly, in performed studies decreased *TES* gene expression associated with HMT of CpG islands nor LOH of chromosome 7q31 was found [3, 19, 25]. In addition, methylation of *TES* promoter region was described in various tumor types. Tobias et al. showed methylation of the CpG islands at the 5′ end in many types of tested tumor-derived cell lines [26]. Tatarelli et al. found fully methylated *TES* promoter in 1/10 breast, 1/8 pancreatic and 9/18 leukemia cell lines [19]. According to Ma et al. methylation of CpG in the *TES* promoter inactivate gene. Moreover, it was revealed that treatment with 5-aza-2’deoxycytidine (DAC), inhibitor of DNA methyltransferase activity, switched completely methylated *TES* promoter into partially or even fully unmethylated region in gastric cancer cell lines [3]. Upregulation of *TES* gene expression after treatment with DAC in glioblastoma cells confirmed that HMT play significant role in *TES* regulation, being responsible for gene silencing [27]. Only one study presented contrary results. Han et al. indicated overexpression of *TES* in GTL-16 gastric cancer cell line [28]. High frequency of LOH was found at 7q31 region in primary gastric cancer, they identified D7S486 to be the most frequent LOH locus [3]. As it was anticipated, there is an evidence presence of LOH in 7q31.2 in many types of neoplasms, e.g. ovary, breast, colorectal, gastric, head and neck, prostate, thyroid, pancreatic and kidney cancer as well in leukemias [29–37]. Ma et al. presented correlation between LOH presence and lack of testin protein expression in gastric cancer [1, 3]. Results are unequivocal as Chene et al. did not disclose such correlations in prostate cancer [4].

### Testin - Epigenetic Modifications, Copy Number Alterations and Mutations Based on Available Database (GDC Data Portal, NGS Data, GEO)

**Fig. 2:**
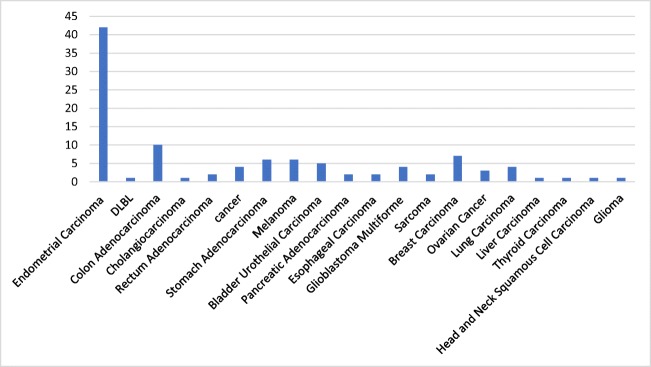
Number of cases and type of cancer affected by TES mutations

According to GDC Data Portal which analyzed 10,202 cases they identified 105 cases with different type of cancers (Fig. 2).

**Fig. 3:**
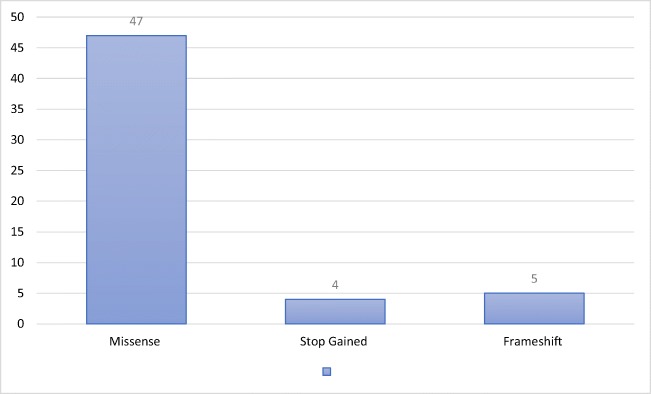
Shown the incidence of types of mutations with a predominance of missense mutations (47)

Analyzed patients was affected by 114 mutations of TES gene described in 21 projects. The largest number of cases was covered by endometrial carcinoma (42 cases), colorectal cancer (12) and breast cancer (7). During the process of transcription was identified missense, stop gained and frameshift mutations (Fig. 3).

The most frequent type of somatic mutation was substitution, described in 91 cases. Two authors cloned and described human TES gene. Tatarelli et al. determined that inactivation of TES is caused by methylation of CpG islands and revealed 3 missense mutation in 26 tumor cell lines [19]. In 2001 Tobias et al. showed also frequent occurrence of methylation of the CpG island at the 5-prime end in ovarian cancer and tumor-derived cell lines [26]. Research on TES gene revealed that TES gene may represent tumor supressor gene.

### Role of Testin in Cancers

#### Ovarian Cancer

According to Knudson^‘^s hypothesis, HMT can be the “second hit” in tumor suppressor genes inactivation [38]. Previous studies showed also high frequency of *TES* gene HMT in the cancer cells [26]. Upregulation of *TES* gene by DAC in ovarian cancer cell line induces cell apoptosis and reduces colony formation preventing from rapid grow of cancer cells [1, 19, 27]. Qui et al. analyzed *TES* expression in regard HMT and LOH using microsatellite analysis and methylation-specific PCR (MS-PCR) in epithelial ovarian cancer cell lines (SKOV3 and A2780) and ovarian cancer samples. Additionally, they demonstrated by immunohistochemistry (IHC) weak testin expression in cancer samples and strong in normal ovarian tissues [1]. Mentioned study is the only one describing role of testin gene and protein in ovarian cancer. Authors did not correlate disclosed expressions with clinicopathological data.

#### Breast Cancer

Zhu et al. conducted studies analyzing association between *TES* gene expression and cell migratory potential tested by Transwell chamber assay as well their invasiveness (expression of matrix metalloproteinase-2; MMP-2) and angiogenesis (expression of CD34, marker of angiogenesis) by IHC. In the perspective of recognized markers they correlated with them *TES* expression. It seems that *TES* play important role not only in tumor formation but also in angiogenesis and metastasis. *TES* gene expression inversely correlates with expressions of MMP-2 and CD34 [24, 39, 40]. Expression of MMP-2 is regulated by miR-29b. Inhibition of MMP-2 through miR-29b can suppress tumor invasion, angiogenesis and metastasis [41]. Interaction between MMP-2 and miR-29b may be useful as therapeutic target for breast cancer (BC). Sarti et al. studied IHC expression of testin in BC patients in a view of their molecular subtype (luminal A, luminal B, basal-like called triple-negative and normal-like BC). Expression of testin was decreased in 74.7% of studied samples, whereas statistically significant downregulation of testin expression was observed in triple-negative and luminal B subtypes. Furthermore, frequency of HMT of CpG in testin varied between BC subtypes and was the highest in luminal B subtype [42]. Consequently, a different correlation levels between testin protein vs. triple-negative and luminal B subtypes may be the result of different grades of HMT of CpG in *TES* promoter region [6, 19, 26]. Low level of testin protein correlates with higher grades of histological malignancy and is unfavorable prognostic marker [25]. Zhu et al. demonstrated in breast cancer (BC) correlation between low *TES* gene expression and shortened survival rates e.g. breast relapse-free, cause-specific, distant metastasis free and overall survival [21].

#### Endometrial Cancer

Dong et al. showed loss of *TES* gene expression in endometrial tissue using PCR. They hypothesized HMT as a main regulator of *TES* expression. Overexpressed *TES* gene significantly induce apoptosis, reduce cell proliferation and arrest cells in G1 phase of cell cycle [2]. Additionally, Gu et al. measured *TES* gene expression by PCR in five endometrial cancer cell lines (AN3CA, Ishikawa, KLE, ECC-1, HEC-1A). They presented influence of *TES* gene on MMP-2. which may control cellular invasion [20]. On the other hand in cancer progression important role plays epithelial-mesenchymal transition (EMT). During this process biology and structure of epithelial cells is switched into features found in mesenchymal ones, i.e. cells lose their polarity, cell-cell adhesion and gain migratory and invasive properties [43]. Downregulation of *TES* gene was observed with decreased expression of epithelial marker- E-cadherin and increased expression of mesenchymal markers: N-cadherin, vimentin and snail. Presence of EMT markers expression significantly correlated with poor outcomes and invasiveness of cancer cell [2]. Immunoexpression of testin was various in different clinical stages and histological grades. IHC analysis revealed decreased testin expression in endometrial cancer cases compared to the adjacent normal endometrium. Decreased expression of testin protein correlated with advanced tumor stage, high grade and lymphatic vascular space invasion. No correlation between testin protein expression and patient age, pathologic types, myometrial invasion and lymphatic metastasis was shown [20].

#### Colorectal Cancer

Li et al. described decreased expression of testin gene and protein in colorectal cancer (CRC). Additionally, they pointed correlation between low testin gene and protein expression and cell migratory as well invasive properties [5]. A disabled apoptotic response may be a major contributor of tumor growth, however mechanism in which testin reduce proliferation and induce apoptosis is still unclear. Study on CRC revealed that high expression of *TES* gene correlate with decreased levels of the anti-apoptotic proteins such as Bcl-2, survivin and increased levels of pro-apoptotic proteins i.e. p53, Puma, Bax. In pathogenesis of sporadic CRC take part uncertain communication between cells which is altered by deviation in p38 mitogen-activated protein kinase pathway (p38-MAPK) [5, 44]. Studies on the role of p38 in CRC cancerogenesis are divergent. Several reports described role of p38 in cell survival and invasion in advanced tumor types whereas involvement in induced cell cycle arrest, differentiation and apoptosis was shown [45, 46]. Western blot (WB) analysis performed by Li et al. on CRC cell lines overexpressing *TES* showed increased p38 phosphorylation. Moreover, inhibition of p38 by specific inhibitor of p38-MAPK (SB203580) markedly promoted proliferation and inhibited apoptosis of cancer cells [5]. This result indicate that activation of MAPK through phosphorylation of p38 in CRC with high expression of *TES* gene is associated with anti-proliferative and pro-apoptotic effect [3].

#### Gastric Cancer

Ma et al. analyzed expression of *TES* gene in gastric normal and cancer cell lines. They comprehensive analysis did not show any specific *TES* gene mutation. However, HMT of CpG islands observed in the region of *TES* gene resulted in downregulation of *TES* gene and protein level in primary gastric cancer [3]. In another study, WB and IHC analysis showed that low protein level was associated with lower differentiation However, there was no relationship with age, sex, tumor size, metastasis, lymphatic invasion and clinical stage. Patients with positive *TES* expression have longer survival time [47].

#### Lung Cancer

The role of testin in lung cancer is not widely described. The only work by Wang et al. describe weak *TES* gene and protein expression in non-small cell lung cancer (NSCLC) cell lines in regard to normal bronchial epithelial cells [46]. The authors explored suppressive effect of testin on proliferation, invasion and colony forming of NSCLC cells. Flow cytometric analysis revealed induced apoptosis in NSCLC cells overexpressing *TES*. Additionally, they presented inhibitory effect of TES on NSCLC cell xenograft formation and growth in vivo on athymic nude mice. These data suggest important role of testin in development and progression of NSCLC [48].

#### Prostate Cancer

*TES* gene is localized at 7q31 region which encodes also others candidate tumor suppressor genes such as *CAV2, CAV1, MET, CAPZA2, ST7, WNT2* [19]. Quantified analysis by RT-PCR displayed that only *TES* gene showed decrease expression in all types of prostate tumors, supporting tumor suppressor gene hypothesis. This is in concordance with results of Tobias et al. who showed reduced growth potential in ovarian (OVCAR5) and cervical (HeLa) cancer cell lines with reduced *TES* gene expression. Chene et al. demonstrated decreased expression of *TES* in confined prostate tumors, tumors with extracapsullary extension and hormonal refractory prostate tumors. However, in hormonal refractory tumors *TES* gene expression was lower than in other types of prostate cancer [24]. Additionally, *TES* gene expression was higher in prostatic epithelial cell lines than in primary prostatic fibroblast [4]. However, results of study by Chene et al. did not find correlation between LOH and *TES* gene expression [26].

#### Head and Neck Squamous Cell Cancer

Gunduz et al. analyzed *TES* expression in regard to clinical advancement (i.e. tumor, lymph nodes and metastasis status; TNM) and survival ratio in head and neck squamous cell cancers. They presented no association between TNM stage and *TES* gene expression, whereas worse survival rate (50% vs. 80%) was observed in cases with weak compared to normal and high *TES* expression [8]. In Li et al. work expressions of testin gene and protein were measured by PCR, WB and IHC. They disclosed lower expression of testin protein in the nasopharyngeal cancer in comparison to the normal tissue. Moreover, protein expression positively correlated with lymph node and distant metastasis and differentiation grade. This protein may be useful as a prognostic tool reference to metastasis in nasopharyngeal cancer [9].

## Conclusion

Various studies showed that *TES* gene silencing can contribute to cancerogenesis revealing its nature of tumor suppressor gene. This findings may be useful in individualized therapy. Understanding molecular mechanisms of cancerogenesis are an important step forward in expanding possibilities of treatment, e.g. usage of 5-aza-2’deoxycytidine. Moreover, there is an evidence suggesting possible usage of testin protein as a prognostic marker. Further studies are necessary to reveal, evaluate and confirm various interesting clinical implications.

### Availability of Data

The datasets analysed in the current study are available on the in the Genomic Data Commons (GDC) Data Portal at https://gdc.cancer.gov/.
